# Genomic signatures of human and animal disease in the zoonotic pathogen *Streptococcus suis*

**DOI:** 10.1038/ncomms7740

**Published:** 2015-03-31

**Authors:** Lucy A. Weinert, Roy R. Chaudhuri, Jinhong Wang, Sarah E. Peters, Jukka Corander, Thibaut Jombart, Abiyad Baig, Kate J. Howell, Minna Vehkala, Niko Välimäki, David Harris, Tran Thi Bich Chieu, Nguyen Van Vinh Chau, James Campbell, Constance Schultsz, Julian Parkhill, Stephen D. Bentley, Paul R. Langford, Andrew N. Rycroft, Brendan W. Wren, Jeremy Farrar, Stephen Baker, Ngo Thi Hoa, Matthew T.G. Holden, Alexander W. Tucker, Duncan J. Maskell, BRaDP1T Consortium, Janine T. Bossé, Yanwen Li, Gareth A. Maglennon, Dominic Matthews, Jon Cuccui, Vanessa Terra

**Affiliations:** 1 Department of Veterinary Medicine, University of Cambridge, Cambridge CB3 0ES, UK; 2 Department of Molecular Biology and Biotechnology, University of Sheffield, Sheffield S10 2TN, UK; 3 Department of Mathematics and Statistics, University of Helsinki, Helsinki 00100, Finland; 4 MRC Centre for Outbreak Analysis and Modelling, Department of Infectious Disease Epidemiology, Faculty of Medicine, London W2 1PG, UK; 5 Department of Computer Science, University of Helsinki, Helsinki 00100, Finland; 6 The Wellcome Trust Sanger Institute, Wellcome Trust Genome Campus, Hinxton, Cambridge CB10 1SA, UK; 7 Oxford University Clinical Research Unit, Wellcome Trust Major Overseas Programme, Hospital for Tropical Diseases, Quan 5, Ho Chi Minh City, Vietnam; 8 Hospital for Tropical Diseases, Quan 5, Ho Chi Minh City, Vietnam; 9 Centre for Tropical Medicine, Nuffield Department of Medicine, University of Oxford, Oxford OX3 7LJ, UK; 10 Department of Global Health—Amsterdam Institute for Global Health and Development, Academic Medical Centre, University of Amsterdam, Amsterdam 1100 DE, The Netherlands; 11 Section of Paediatrics, Faculty of Medicine, Imperial College London, London W2 1PG, UK; 12 The Royal Veterinary College, Hawkshead Campus, Hertfordshire AL9 7TA, UK; 13 Faculty of Infectious & Tropical Diseases, London School of Hygiene & Tropical Medicine, London WC1E 7HT, UK; 14 A full list of consortium members appears at the end of the paper.

## Abstract

*Streptococcus suis* causes disease in pigs worldwide and is increasingly implicated in zoonotic disease in East and South-East Asia. To understand the genetic basis of disease in *S. suis*, we study the genomes of 375 isolates with detailed clinical phenotypes from pigs and humans from the United Kingdom and Vietnam. Here, we show that isolates associated with disease contain substantially fewer genes than non-clinical isolates, but are more likely to encode virulence factors. Human disease isolates are limited to a single-virulent population, originating in the 1920, s when pig production was intensified, but no consistent genomic differences between pig and human isolates are observed. There is little geographical clustering of different *S. suis* subpopulations, and the bacterium undergoes high rates of recombination, implying that an increase in virulence anywhere in the world could have a global impact over a short timescale.

## Abstract (web summary)

The bacterium *Streptococcus suis* causes respiratory tract infections in pigs and meningitis in humans. Here, the authors show that human disease isolates are limited to a single virulent population and find no consistent genomic differences between pig and human isolates.

## Graphical abstract

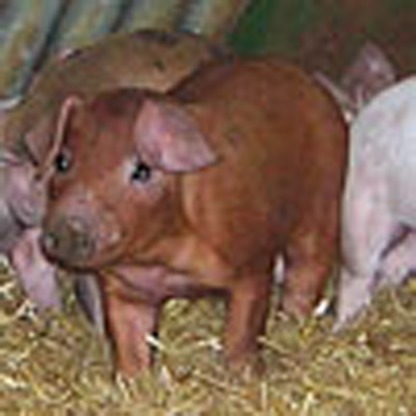

The bacterium *Streptococcus suis* colonizes the upper respiratory tract of pigs asymptomatically, but it can also cause respiratory tract infections as well as serious invasive diseases such as arthritis, septicaemia and meningitis. These diseases are responsible for major worldwide economic losses to the pig industry as well as being important drivers of antibiotic prophylaxis to large groups of pigs. *S. suis* can also infect humans, and recently it has become a serious endemic public health threat, being the most common cause of adult bacterial meningitis in parts of South-East Asia^1^. *S. suis* has also been responsible for zoonotic outbreaks in China, with high mortality rates associated with a Streptococcal Septic shock-like syndrome^2^. Sporadic outbreaks of this kind are a particular public health concern because they represent a major source of emerging disease with potential global consequences.

Infection of humans with *S. suis* appears to result from prolonged or repeated close contact with infected pigs or their products, and epidemiological surveillance has identified a number of risk factors, including the consumption of undercooked pig tissues^3^. Furthermore, despite limited evidence of carriage in human tonsils^3^, *S. suis* does not appear to be easily transmitted between humans. Nevertheless, it is possible that the recent increase in *S. suis* zoonoses was facilitated by the genetic adaptation of *S. suis* to its human host, as has been observed in other pathogens such as methicillin-resistant *Staphylococcus aureus* (MRSA) ST97 (ref. 4). In particular, human *S. suis* cases are mainly associated with serotypes 2 and 14, although there are reports of single cases associated with serotypes 1 (ref. 5), 4 (ref. 6) or 16 (ref. 7) implying some degree of host specificity, and thus pathogen adaptation. But, despite its clear importance for public health, little is currently known about the genetic changes that distinguish serotypes 2 and 14 from other serotypes, or whether any other genetic changes are associated with the isolates from human patients.

Here, we investigate the evolution of *S. suis* using whole-genome sequencing combined with high-quality clinical data and focused sampling to lessen the impact of confounding environmental factors. Specifically, to address the evolution of swine disease, we have sampled 184 isolates from pigs from across the United Kingdom, including large numbers of carriage isolates from pigs with no clinical signs, as well as isolates from documented cases of respiratory and systemic disease. To address the evolution of human disease, we sampled 191 isolates from humans and pigs from across Vietnam, the source of the most recent and serious zoonoses.

We find significant genomic differences between non-clinical isolates from the upper respiratory tract, isolates causing respiratory disease and isolates causing systemic disease. In addition, disease-causing isolates contain substantially fewer genes than non-clinical isolates, but despite this genomic reduction are more likely to have acquired genes encoding known virulence factors. We also identify additional virulence candidates, in particular, two genomic regions on a mobile element correlating with respiratory disease. However, we do not find any genomic differences between human and pig hosts, although human disease isolates are limited to a single virulent population whose origin coincided with the first intensification of pig production.

## Results

### Global diversity of *Streptococcus suis*

We sequenced 191 isolates of *S. suis* from Vietnam and 184 from the United Kingdom. To contextualize these isolates within the global diversity of *S. suis*, we combined our data with 15 published *S. suis* genome sequences and drafted genomes sampled from North America, South America, Europe and Asia, giving a total data set comprising 459 isolates (see Supplementary Data 1 for details of isolates and references).

To investigate the genetic structure of the collection, we extracted the sequences of the core genome and used a Bayesian clustering method (BAPS) to identify five distinct populations (Supplementary Fig. 1). Consistent with findings in other streptococci^8^, there was evidence of extensive genetic recombination between and within populations (Supplementary Fig. 2), preventing us from reliably inferring the evolutionary relationships between the populations. Interestingly, recombination relative to point mutation (*r/m*) values are higher in non-clinical (3.81) compared with clinical (1.19—systemic and 1.9—respiratory) isolates.

**Figure 1:**
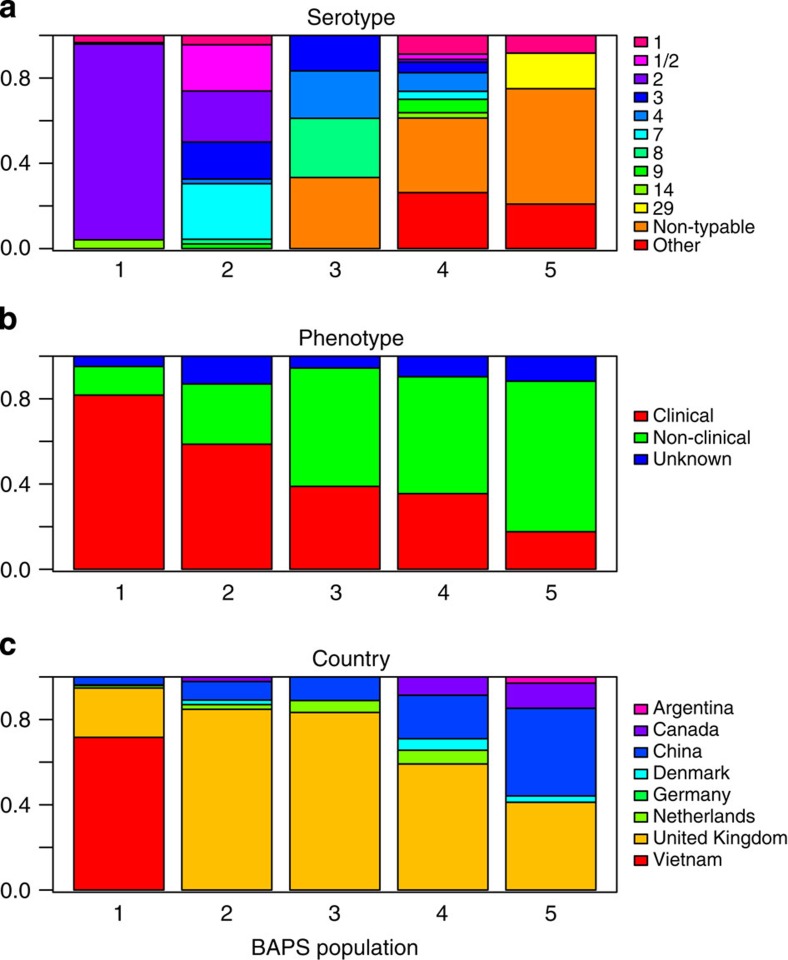
*S. suis* populations broken down by metadata. The distribution of (**a**) serotype, (**b**) clinical phenotype and (**c**) country of origin, among the five populations of *S. suis* that were identified by our Bayesian analysis of population structure. The figures show that there is little genetic structure with regard to the metadata, although a partial exception is population 1, which is dominated by isolates from Vietnam, and serotype 2, and which contains all the zoonotic isolates.

Comparison of the genetic structure defined by the Bayesian clustering and metadata associated with each isolate (Fig. 1) showed relatively little structure associated with serotype or the clinical outcome in the pig (Fig. 1a,b). For example, serotype 2 dominates population one, but most serotypes were found in multiple populations. Similarly, all five populations contained both non-clinical and disease-causing isolates (though there was variation in proportions, with population 1 showing the largest number of disease-causing isolates). Together, these results indicate high levels of capsule switching (which determines serotype) among different genetic backgrounds (Fig. 1a) and substantial evolutionary lability in pathogenicity (Fig. 1b).

Examination of the distribution of sampling locations showed little evidence of genetic structure. In particular, isolates from the United Kingdom were found in all five populations, as were those from China (Fig. 1c). Isolates from several other countries (which were less densely sampled) were also found in multiple populations. The analysis therefore suggests full global admixture of *S. suis*. Notable exceptions are the isolates from Vietnam, which were selected because of their serotype (serotype 2) and were assigned to population one. In addition, all known isolates from humans from China (4 isolates), The Netherlands (1 isolate) and Vietnam (154 isolates) are found within this population. This suggests that a subset of the genetic diversity of *S. suis* might be more capable of causing human disease, in accordance with recently published data^9^,^10^.

### Differences between clinical and non-clinical pig isolates

**Figure 2:**
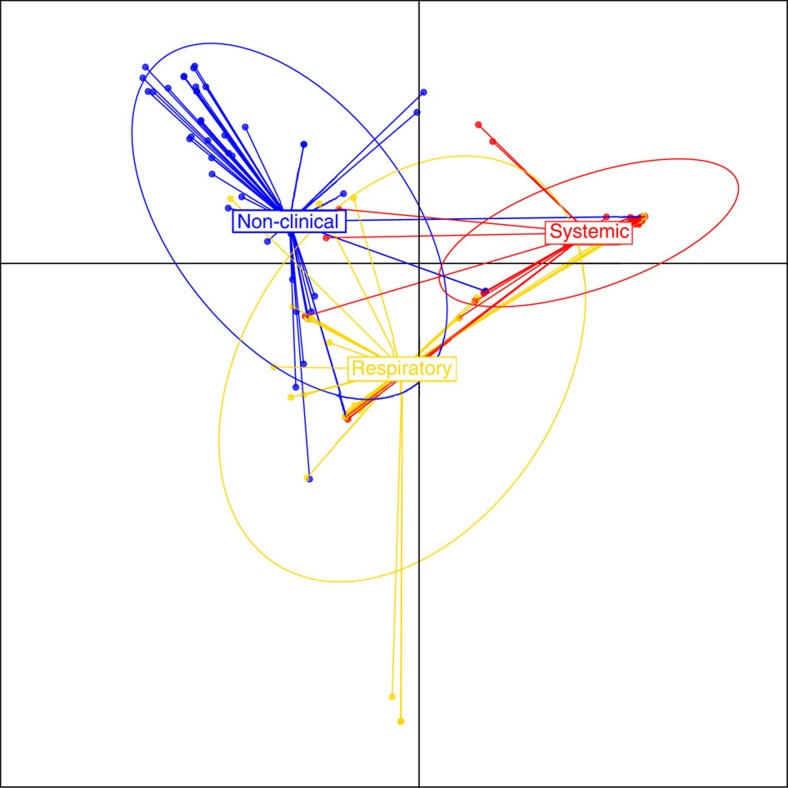
Genomic differences between non-clinical, systemic and respiratory strains. Discriminant analysis of principal components, applied to 153 *S. suis* isolates isolated from pigs in the United Kingdom, using SNPs in the core genome, and presence/absence data for genes in the accessory genome. Isolates were classified as non-clinical (isolated from the nasal passage), systemic (disease-causing and isolated from the brain, blood or joints) or respiratory (disease-causing and isolated from the lungs).

To investigate the genetic basis of swine disease, we focused on 153 of the 184 isolates sampled from pigs in the United Kingdom, which are associated with a well-defined clinical phenotype (see methods). Of these, 62 were isolated from nasal carriage and non-clinical animals, 39 were isolated from the lungs and associated with a respiratory-type illness, and 52 were isolated from the blood, joints or brain and associated with systemic illness (Supplementary Data 1). Using discriminant analysis of principle components (DAPC)^11^ we found clear genetic differences in the combined core and accessory genomes associated with these phenotypes. Figure 2 shows the first and second linear discriminant functions between non-clinical, respiratory and systemic isolates. There is a clear group separation between non-clinical and systemic isolates with very few exceptions. The respiratory isolates are also genetically distinct from both systemic and non-clinical isolates, but include many more isolates that overlap both of the other groups.

### Disease-causing isolates have smaller genomes

**Figure 3:**
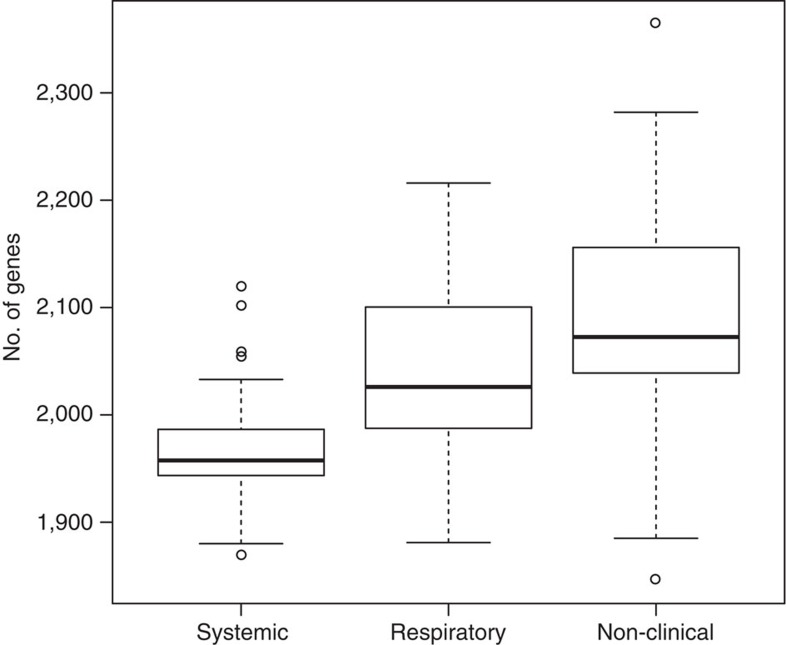
Genome size differences in isolates of different clinical phenotype. The number of genes in 153 *S. suis* isolates isolated from pigs in the United Kingdom. Isolates were classified as non-clinical (isolated from the nasal passage) *n=62*, systemic (disease-causing and isolated from the brain, blood or joints) *n=52* or respiratory (disease-causing and isolated from the lungs) *n=39*. Boxes show the medians and upper and lower quartiles; whiskers show the most extreme values within 1.5 times the interquartile range.

A second striking difference was found in the genome sizes of the different isolates (Fig. 3). Systemic isolates had around 50 genes fewer (on average) than respiratory isolates, and these had around 50 genes fewer (on average) than the non-clinical isolates. This observation appears not to be confounded by population structure, because it holds in each of the three populations that contain several non-clinical and systemic isolates from the United Kingdom (populations 2–4; Supplementary Fig. 3).

While the pattern of genome reduction was consistently associated with pathogenicity, we found no clear pattern in the types of genes that were absent. In particular, there was little difference in the distribution of functional classes between the genes that were absent from all systemic isolates, and those that were present (Supplementary Fig. 4). We therefore investigated cases of repeated evolutionary loss (or repeated gain by non-clinical isolates) by analysing individual genes that were consistently less prevalent in systemic isolates from *S. suis* populations 2–4 (the populations with the greatest mixture of clinical and non-clinical isolates). Only one gene, a pyruvate synthase, was present in all three populations but absent from all of the systemic isolates from this study. However, 45 genes (Supplementary Data 2) were consistently under-represented in systemic isolates, of which five are linked in the pan genome. These five genes appear to encode phage proteins and exhibit homology to those found in a previously described lytic bacteriophage of *S. suis*^12^. Of note, this list also contains many transcriptional regulators, and this same pattern of loss of regulatory complexity leading to pathogenicity has been observed in other bacteria^13^.

### Virulence genes are over-represented in systemic isolates

**Figure 4:**
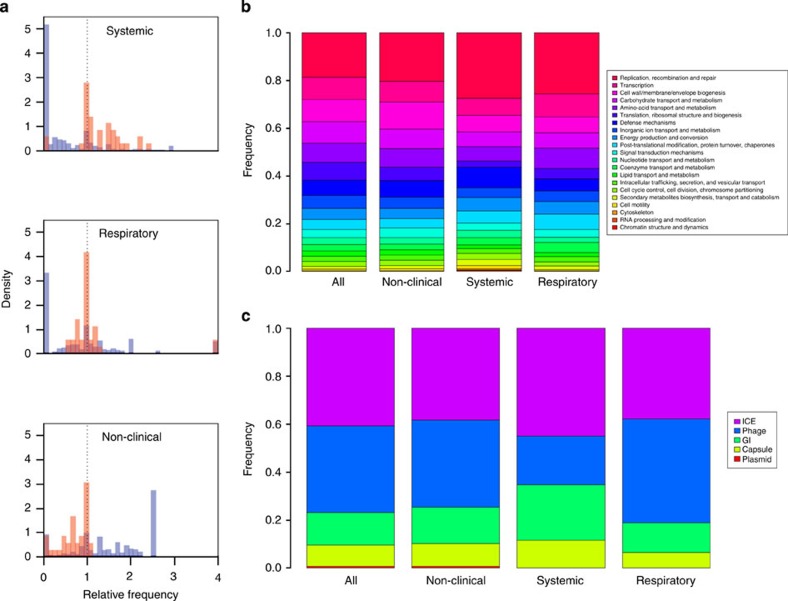
Differences in the accessory genome content of 153*S. suis* isolates isolated from pigs in the United Kingdom. Isolates were classified as non-clinical (isolated from the nasal passage), systemic (disease-causing and isolated from the brain, blood or joints) or respiratory (disease-causing and isolated from the lungs). (**a**) Shows a histogram of the relative frequencies of known virulence genes (orange) versus all other genes (blue). For the systemic isolates, ‘relative frequency’ is defined as *p*_sys_*/p*_tot_, where 0<*p*_sys_<1 is the proportion of systemic isolates in which the gene appears, and *p*_tot_ is the same quantity for the total 153-isolate data set. (**b**) Shows the functional categories associated with all accessory genes, as compared with the subset of these genes that are over-represented in each category of isolates (defined as genes whose relative frequency is >2). (**c**) Shows the presence on known MGEs for the genes shown in (**b**).

Despite the consistent pattern of genome reduction, certain categories of gene were present more often than expected in systemic isolates. In particular, there was a clear tendency for genes encoding known or putative virulence factors^14^ to be over-represented in systemic isolates (Kruskal–Wallis rank sum test of mean relative frequencies defined in the legend of Fig. 4; *χ*^2^=57.1138, *n*=52, *P*=4.113 × 10^−14^), and under-represented in non-clinical isolates (χ^2^=40.8519, *n*=62, *P*=1.642 × 10^−10^), while most other genes showed the opposite pattern (Fig. 4a). In respiratory isolates, the pattern was intermediate, with virulence genes being present at a slightly higher frequency but the *P* value was just greater than the arbitrary 5% threshold for statistical significance (*χ*^2^=3.6448, *n*=39, *P*=0.05624). These patterns did not differ between known and putative virulence factors^14^, suggesting that the attributions are accurate.

Of particular interest are two virulence genes that show an anomalous pattern, being completely absent from the systemic isolates (Fig. 4a). These two genes (*salK*/*salR*) make up a two-component signal–transduction system found on a mobile genetic element (MGE), an 89 kb pathogenicity island, which has been linked to high virulence of Chinese isolates in an animal model^15^. Despite being absent from the systemic isolates from the United Kingdom, they are found in many of the Vietnamese isolates in our collection (some dating back to 2000) and are also found within respiratory and non-clinical serotype 4 isolates from the United Kingdom. Closer inspection showed that the genes within serotype 4 are divergent from the genes found within the serotype 2 isolates from East Asia (Supplementary Fig. 5).

Equally intriguing is the small number of ‘control genes’ whose patterns of presence and absence mirror those of the known virulence factors. Figure 4b shows the functional classes of these genes that are substantially over-represented in each category of isolate. Disease-causing isolates tend to have a greater number of genes involved in replication, recombination and repair but, surprisingly, fewer genes involved in cell wall and membrane biogenesis. In addition, systemic isolates have a larger number of genes involved in defence functions. Figure 4c shows the location of these genes on MGEs (identified MGEs from the pan genome shown in Supplementary Fig. 6). Genes over-represented in systemic isolates are more likely to be found on the integrative conjugative element (ICE), genomic islands and the capsule locus but are much less likely to be phage-associated.

Lastly, we investigated repeated evolution of pathogenicity by focusing on genes that were consistently over-represented in systemic and respiratory isolates in the three populations with the greatest mixture of non-clinical and clinical cases (populations 2–4). We detected 12 genes that were consistently over-represented in systemic isolates, although no discernible pattern could be found linking these genes to functional pathogenicity (Supplementary Data 3). A few of the genes are found on the ICE but they are not found within the same region. One capsule gene was identified, which could be important for host interaction, but this bears little correlation with serotype being found in serotypes 1, 3, 4, 7 and in non-typeable isolates.

Conversely, we identified 47 genes that were consistently over-represented in respiratory isolates (Supplementary Data 4). Many of these were found to be linked together, either functionally, for example, the 6 capsule-associated genes almost exclusively associated with serotype 3 isolates, or proximally in the case of 26 of the 47 over-represented genes that are found on a horizontally transferred 48 kb region. In many of the isolates, this region is located next to an ICE and contains genes encoding an IgA-specific *zinc metalloproteinase* gene (*zmpC*; Supplementary Fig. 7) and putative fucose utilization operon including a glycoside hydrolase family 98 protein (Supplementary Fig. 8), both of which have been linked to virulence in *S. suis* and other streptococci. The *IgA1 protease* gene is important for pathogenesis in *S. suis*^16^, and a *zmpC* gene plays a role in pneumococcal disease in *S. pneumoniae*^17^. In addition in *S. pneumoniae,* the fucose utilization operon has been linked to colonization and virulence^18^,^19^, suggesting a role in respiratory infection. Analysis of the distribution of the 48 kb ICE-associated region in the 153 UK pig isolates shows that it is present in 21 isolates that form a monophyletic group (in a tree stripped of recombination events) in the population. This region is also present in the isolates from Vietnam and China, suggesting a wider dissemination for this pan genomic component.

### Emergence of the zoonotic clade in the 20th Century

**Figure 5:**
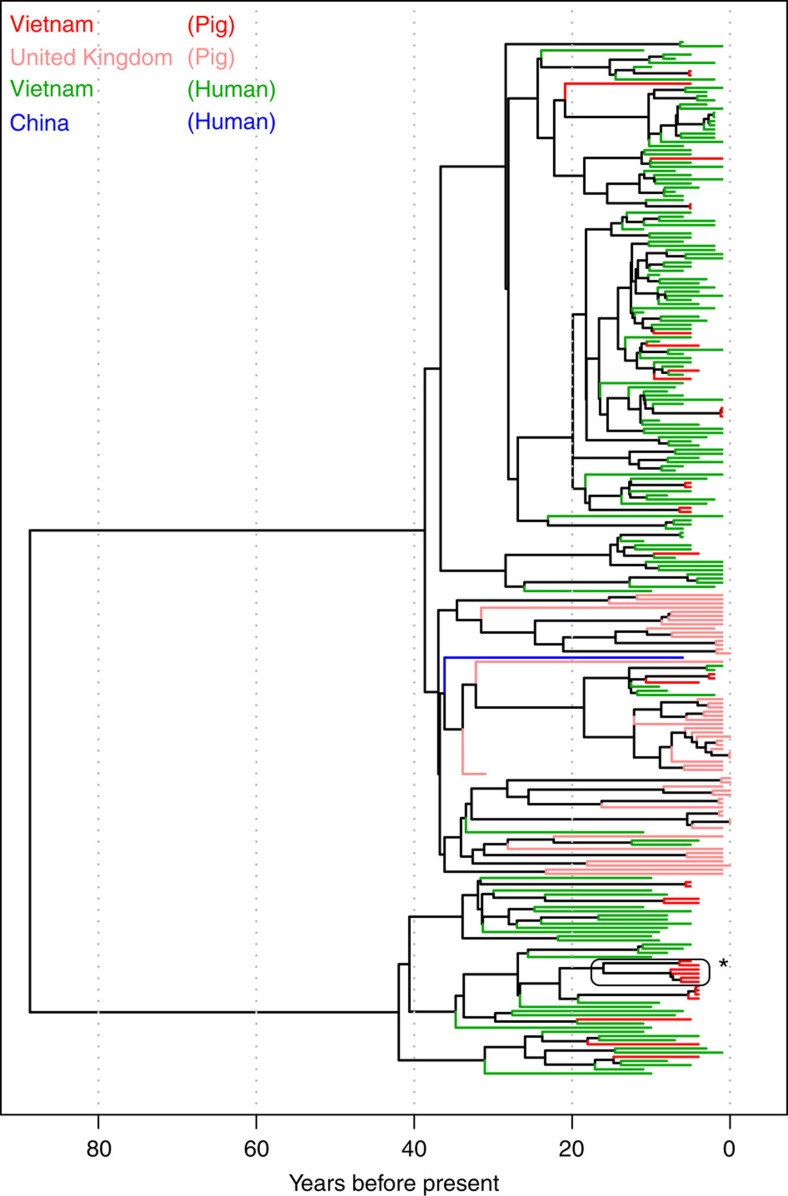
Dated phylogeny of a virulent zoonotic clade of *S. suis*. Phylogeny shows 256 isolates of *S. suis* from virulent population one that contains all of the zoonotic isolates. Terminal branches are coloured according to the country and host from which the isolate was obtained, and indicate some genetic structuring by country, but little clustering by host. The phylogeny is a Maximum Clade Consensus tree, estimated from an expanded core genome, with MGEs and recombinant sites removed. The clade denoted with an asterisk corresponds to the isolates shown in the right hand peak of Fig. 6.

Population one was associated with the highest proportion of pig disease-causing isolates, and with all of the zoonotic isolates. We therefore investigated the ecological context of the emergence of this virulent zoonotic population. To do this, we compiled an extended core genome for this population, and after stripping recombinant sites, used a relaxed molecular clock and the sampling dates of our isolates (range 1980–2011) to date its origin. The dated phylogeny is shown in Fig. 5. Two distinct clades are evident, with some geographical structuring between the UK and Vietnam isolates in one of the clades. The origin of this virulent group is estimated to be 1921 (95% Highest Posterior Density: 1901–1938), with each of the clades arising in the 1970, s. Furthermore, we found evidence of rapid population expansion of this group (with an exponential coalescent growth model favoured over a constant growth model; Bayes Factor=28), although growth rates can be overestimated when using data that have been stripped of recombination, as is the case here^20^.

### No evidence of host adaptation to humans in Vietnam

**Figure 6:**
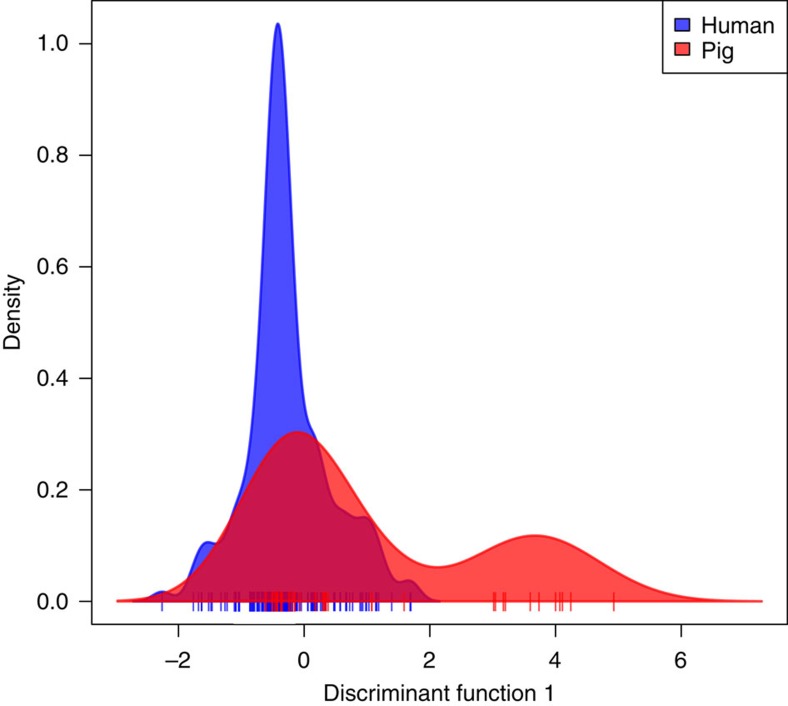
No genomic differences between isolates from human and pig hosts in Vietnam. Discriminant Analysis of Principal Components, applied to 191 isolates isolated from human and pig hosts in Vietnam, using SNPs in the core genome, and presence/absence data for genes in the accessory genome. Shown is the first linear discriminant function, and the lack of separation between the distributions suggests a lack of consistent genetic differences between isolates from the two host types.

Figure 5 shows that the 191 isolates from Vietnam show little phylogenetic structuring by host, with the pig samples scattered throughout the tree. Nevertheless, it is possible that genetic variants, associated with human pathogenicity, have evolved multiple times, either by convergent molecular evolution or by gene exchange. To ask whether any genetic variants were consistently associated with pig or human isolates, we used three different methods. For associating single-nucleotide polymorphisms (SNPs) in the core genome, we used the software PLINK^21^. However, since adaptive genomic change might be found in both the core genome and the accessory genome, we used DAPC by examining biallelic SNPs from the core genome and the presence or absence of accessory genes, ordered by synteny, and a k-mer analysis, which examines associations between DNA ‘words’ of 10–100 base pairs and host 22 (see online methods). After controlling for bacterial population structure, bacterial phylogeny and after correcting for multiple tests (see online methods), we did not detect any core genome SNP associated with human hosts. However, we did detect one significant core genome SNP that was polymorphic in pig hosts while found at low frequency in human hosts (Cochran-Mantel-Haenszel test, *n*=191, *P*=0.001). This SNP represents a non-synonymous change in a 16 S rRNA methyltransferase family protein. The first linear discriminant function of the DAPC is shown in Fig. 6, and shows that the genetic variants give us little power to classify isolates as being of pig or human origin, although there is a population of *S. suis* that is found in pigs but not humans (Fig. 6; right hand peak). These unique pig isolates can be traced to one clade of non-clinical pig isolates on the phylogeny (indicated by a star Fig. 5). The most discriminatory sites that explain this differentiation can be traced to genomic island 6 since these pig isolates lack this island. However, many human isolates also lack this element (Supplementary Fig. 9), so this cannot explain this non-pathogenic phenotype. Finally, none of the k-mers found in the isolates were significantly associated with human hosts. Taken together, there are no genetic variants (SNPs or accessory genes) that are substantially more likely to appear in isolates from human infections, indicating little or no consistent adaptation to the human population.

## Discussion

Using high-quality clinical data and whole-genome sequencing, we have shown clear genetic differences between systemic, respiratory and non-clinical (carriage) *S. suis* isolates. This is somewhat surprising given that systemic isolates are thought to infect pigs through the nasopharynx and so can occupy the non-clinical ‘niche’. Therefore, one might expect some isolates phenotypically defined as non-clinical to be capable of becoming systemic, but the observed genetic differentiation implies that disease outcome is more genetically deterministic. In contrast, respiratory isolates showed more overlap between non-clinical and systemic populations. This suggests that many non-clinical isolates can lead to clinical respiratory infection, probably dependent on the presence of other environmental factors, such as reduced immunity, lower standards of welfare or co-infection with another pathogen.

Systemic isolates tended to have an over-representation of virulence factors and a larger number of genes involved in defence functions but showed less of a signal of recurrent evolution of specific genes in different populations than respiratory isolates. It is interesting to speculate whether the recurrent evolution of genes relating to function in respiratory isolates but not in systemic isolates relates to the different ecological specialization of these phenotypes, as the respiratory tract is almost certainly more ecologically diverse than systemic locations, which could facilitate the horizontal transfer of mobile genetic elements. Indeed recombination rates are higher in respiratory isolates compared with systemic isolates. In this regard it is notable that our analysis of the pan genome identified a horizontally transferred 48-kb region encoding functions linked to virulence and colonization, which was over-represented in respiratory isolates of pigs.

In addition to gaining virulence factors, disease-causing isolates had significantly smaller genomes than non-clinical isolates. Genome reduction associated with pathogenicity is a pattern observed in many other bacteria^13^. This phenomenon could relate to the presence of ‘antivirulence genes’, which may hinder the full expression of newly acquired virulence factors^23^ or could simply be due to a passive loss of regulatory function that results in uncontrolled multiplication^13^, leading to large increases in bacterial cell numbers within the host.

An adaptive explanation for an increase in bacterial population sizes could be recent changes in the selection pressure for higher bacterial transmission rates. We have shown that the emergence of the virulent zoonotic population dates to the early 20th Century. This coincides with the wide-scale introduction of indoor rearing of meat-producing pigs in larger groups, supported by government schemes that favoured larger producers with regular throughput^24^. In addition, the exponential population size expansion of this clade throughout the 1960 and 1970, s aligns closely to the emergence of the pyramid production methods used for the international dissemination of improved pig genetics. There was major focus at this time on selective breeding of pigs for improved productivity in the United Kingdom and Unitd States at a small number of ‘nucleus’ breeding herds. Gilts and boar semen were then distributed from these nucleus units to ‘multiplication units’ for rapid expansion of the selected pig genetics (and in the case of gilts and live boars, with *S. suis* carried on their tonsils). Health programmes were introduced to support this system, but *S. suis* control was not included in the majority of them. Where *S. suis* was included, there were frequent break-downs as determination of carriage was difficult and usually based on the absence of clinical disease^25^. This pattern of international trade in live pigs may also explain why we observe global admixture of *S. suis* isolates. This trade continues today with Asian markets importing pigs as future breeders from companies in the United States and the European Union.

The global admixture of isolates emphasizes the importance of epidemiological factors in occurrence of human disease—as evidenced by the differences in prevalence of human disease in the EU compared with Vietnam, with associated risk factors. Indeed, we did not find any evidence of consistent genomic adaptation of *S. suis* to the human population.

We also observed high levels of recombination in *S. suis* worldwide. This suggests a potential role for non-clinical isolates—with their larger genomes and correspondingly larger functional repertoire—to act as sources of new adaptive phenotypes that might be transferred to pathogenic isolates. This emphasizes the ongoing importance of including non-pathogenic carriage isolates in efforts to monitor bacterial populations. Finally, this study highlights the value of undertaking microbial genome analysis studies across broader populations that encompass different hosts, geography and, importantly, defined clinical phenotypes.

## Methods

### Sampling of isolates

To investigate the genomic signatures of swine disease, 100 clinical and 100 non-clinical field isolates of *S. suis* from the United Kingdom were isolated by the Animal Health and Veterinary Laboratories Agency (AHVLA) from tissues of pigs submitted from farms in the United Kingdom during 2009 and 2011. These isolates were obtained as part of routine diagnostic investigations on submitted post mortem material and the sources of isolates were anonymized. Isolation of *S. suis* from tissues was by inoculation onto Columbia agar (Oxoid Ltd. Basingstoke, UK) containing 5% (v/v) sheep blood (TCS biosciences Ltd., Bucks, UK) and aerobic incubation at 37 °C for up to 48 h. Suspect *S. suis* colonies (based on colony morphology and alpha-haemolysis) were subcultured, and biochemical confirmation was undertaken on pure cultures (API 32-Strep, Bio-merieux). Antigen extraction for serotyping was performed using Todd-Hewitt broth (Oxoid Ltd. Basingstoke, UK) with autoclave extraction. Serotyping was predominantly performed using antisera to the known *S. suis* serotypes by precipitation using the Lancefield method^26^, where necessary capsule swelling was also performed^27^. Isolates that could not be typed by these methods were classified as non-typeable. Isolates recovered from systemic sites in pigs with clinical signs and/or gross pathology consistent with *S. suis* infection (including meningitis, septicaemia and arthritis) were classified as systemic, whereas those recovered from the lung in the presence of gross lesions of pneumonia were classified as respiratory. Finally, isolates from the tonsils or tracheo-bronchus of healthy pigs or pigs without any typical signs of *S. suis* infection but diagnosed with disease unrelated to *S. suis* (such as enteric disease or trauma) were classified as non-clinical. Isolates for which there was insufficient information about the pigs sampled were classified as unknown.

To investigate whether we see any evidence of adaptation of *S. suis* to humans, human and pig isolates were collected from Vietnam. Five hundred and forty-two pig tonsils were collected from three slaughterhouses in HCMC from September 2006 to November 2007 (ref. 28). Pigs were sent from seven provinces in southern Vietnam and the greater HCMC area. Tonsils were minced and a portion was used to inoculate selective blood agar plates and selective Todd Hewitt broth, which was subcultured on selective blood agar plates after overnight incubation and 32 isolates were obtained from single colonies. Six additional isolates were included, which were isolated from blood of pigs with invasive disease, from the provinces of Soc Trang, Tien Giang and Dong Nai in 2009 and 2010. The study protocol was reviewed and approved by the Sub Department of Animal Health of Ho Chi Minh City (1242/SNNNN). All sampling was performed under the supervision of government staff following standard rendering procedures.

To extensively sample *S. suis* from human hosts, 153 isolates were isolated from cerebrospinal fluid of human meningitis patients, who originated from provinces in southern and central Vietnam, as part of routine practice at the Hospital for Tropical Diseases, Ho Chi Minh City between 2000 and 2010. All strains used in this study were obtained from samples that were collected for diagnostic purposes as part of standard care, at The Hospital for Tropical Diseases (HTD) in Ho Chi Minh City, Vietnam. The study protocol was approved by the ethical committee of the HTD (CS/NĐ/09/13).

### Sequencing

Genomic DNA was prepared from UK isolates grown overnight at 37 °C in Todd-Hewitt broth plus 0.2% yeast (Oxoid Ltd.) using a MasterPure Gram Positive DNA isolation kit (Epicentre). Genomic DNA was prepared from Vietnam isolates grown on sheep blood agar plate using DNeasy blood and tissue kit (Qiagen). The total genomic DNA retrieved varied between 2 to 5 μg, and the qualified DNA samples were those with an OD_260/280_ ratio between 1.8 and 2 using the spectrophotometer (Nanodrop, Thermo scientific).

Samples of genomic DNA (typically 500 ng) were used to prepare multiplexed libraries suitable for sequencing on Illumina instruments^29^,^30^. Sequencing was performed on Illumina HiSeq 2000 instruments operated according to the manufacturer’s instructions with 75 or 100 cycle paired end runs.

### *De novo* assembly

The sequence reads were trimmed for the presence of Illumina adapter sequence using Cutadapt^31^ and quality-trimmed using sickle^32^. An assembly was generated using Velvet version 1.1 (ref. 33), with parameter selection using VelvetOptimiser^34^. Draft genome sequences were annotated using Prokka^35^. Any assemblies with an n50<10,000 were excluded from further analysis. In addition, to check the quality of the *de novo* assembly, the raw reads were mapped back to the assembled isolate and 16 were discarded. Assembly statistics of isolates are shown in Supplementary Data 5.

### Identification of the *S. suis* core genome

We identified the core genome of *S. suis*, considering both our isolates and all published *S. suis* isolates (excluding isolates 89/1591 and 05HAH33 as these assemblies are incomplete; see below). To do this, we extracted all annotated coding sequences (CDSs) from the genome of *S. suis* P1/7 (Genbank Accession: AM946016)^36^ and used these as the query in BLASTN searches against a nucleotide BLAST database of all *de novo* assemblies and complete genome sequences. CDSs that showed >80% identity over at least 80% of the length of the equivalent P1/7 CDS were retained, and the core genome was defined as the genes that were identified in all assemblies using these criteria. Three genome sequences (strains ‘86–5192’, ‘88–1861’, ‘89–4109–1’) from Chen *et al.*^9^ were excluded from the core genome analysis, as including them drastically reduced the number of genes included in the core genome (that is, 675 versus 793). The final number of CDSs in the core genome was 793, the total length was 704,772 bp, the number of SNPs was 178,979 and the final number of taxa was 459.

### Identification of orthologous proteins using OrthoMCL

To reconstruct the accessory genome, we used the program OrthoMCL^37^ to predict homology groups of all *S. suis* genes. CDSs were predicted from each draft genome sequence using default parameters in Prodigal^38^. These predicted CDSs were then extracted and combined with coding sequences extracted from the 14 published *S. suis* complete genome sequences and the draft genome sequence of isolate R61. Two published *S. suis* draft genome sequences (isolates 89/1591 and 05HAH33) were excluded from the analysis after preliminary tests showed many genes missing in these isolates but present in our assemblies and in published complete genome sequences. The draft genomes of these isolates were much shorter in length than all the other isolates, which may reflect low coverage across some regions of the genome. We also excluded draft genome sequences from Chen *et al.*^9^ A BLAST database of the predicted protein sequences was built and the protein encoded by each gene was queried against this database using BLASTP. This all-against-all BLAST list was parsed using the orthoMCLBlastParser Perl script to compute the percent match of each hit^37^. From this, reciprocal best similarity protein pairs were identified by orthoMCL using a percent match cutoff of 50 and a BLAST *e*-value cutoff of 1e−5. Homology groups were then identified by clustering with MCL using different values of an inflation parameter that regulates cluster tightness^39^. At low values, this parameter is more likely to yield false-positive results (that is, where homology groups are clustered together that should be separate) and at high values, true homology groups will become separated. We chose a low value to maximize the number of homology groups with one and only one representative in each of the 390 isolates (that is, core genes), while maintaining a low number of homology groups containing duplicates (that is, homology groups with recent paralogues, that might, therefore, have more than 390 members). The final inflation value chosen was 2.6. The methods applied to identify false positives derived from the use of a low-inflation value are detailed below. All of the resulting 7,675 homology groups of protein-coding genes were then aligned using the Wilbur and Lipman algorithm^40^, and neighbour-joining trees produced using ClustalW^41^,^42^.

### Quality checks of homology groups

Assignment to homology groups can be inaccurate for a number of reasons, including sequencing errors (such as the introduction of erroneous frameshifts, altering many amino acids), assembly errors (such as the mapping of a single ORF to multiple contigs), failure to distinguish between orthologues and paralogues (leading to both over- and underestimation of the number of groups), failure to distinguish between recent duplicates (the presence of multiple copies of a gene), and recent pseudogenes (the presence of multiple segments of the same gene). To address these issues, we manually examined a large number of homology groups. In particular, all groups meeting one or more of the following criteria were manually inspected: (1) groups with slightly more or slightly fewer than 390 members; (2) groups whose members had been assigned multiple, inconsistent annotations; (3) groups without duplicate isolates, with <60% mean amino-acid identity among all members; (4) groups containing duplicate isolates (that is, two or more different genes from the same assembly) and <90% mean amino-acid identity; (5) groups containing duplicate isolates, with <35% sequence overlap; (6) any group containing a gene <80% of the modal length for that group; (7) pairs of groups with high sequence similarity and non-overlapping isolate membership (these groups were identified using reciprocal BLASTP of the longest member of each pair of groups; groups that were reciprocal best BLAST hits and that showed >80% homology over >50% of the length of the amino acid sequence were defined as similar); and (8) groups that showed evidence of containing deep paralogues. For criterion (8), we estimated a neighbour joining tree for each homology group, and used midpoint rooting to infer the two most distantly related clades. *F*_st_ was then calculated to determine the genetic diversity between, as opposed to within, these clades. High *F*_st_ values for clades containing members from overlapping groups of isolates were taken as possible evidence of misidentified paralogues. Each of these tests was carried out using custom R scripts, and 2,276 of the 7,675 groups were checked by eye. For several groups, split CDSs or short sequences (<80% of the modal length for that group^43^) were labelled as pseudogenes and removed from subsequent analyses.

### Identification of virulence genes and functional categories

To identify known virulence genes, we extracted the coding sequence of 71 virulence factors from the list compiled by Fittipaldi *et al.*^14^ and BLASTed them against a database comprising one representative of each of our homology groups. To associate other genes with a functional category, we BLASTed members of each of our homology groups against the COG database^44^.

### Construction of the pan genome

To identify conserved regions of the *S. suis* genome and regions with dynamic gene flux (that is, prophages and integrative conjugative elements), we attempted to syntenically order our homology groups. To do this, we first ordered homology groups containing *S. suis* isolate P1/7 according to the order of genes in the P1/7 genome. Where homology groups contained two P1/7 CDSs (that is, recent paralogues), we took the position of one copy chosen at random. Next, we added in homology groups missing in P1/7 but found in *S. suis* BM407 by placing these homology groups in between genes shared by P1/7 and BM407 according to the order found in the BM407 genome. An illustration of this process is given in Supplementary Fig. 10. We iteratively added in all the *S. suis* complete genomes and then added homology groups on the largest going to the smallest contig. Some homology groups were excluded from this ordered pan genome, as the contigs that they were found on did not contain genes already in the pan genome and thus their position could not be determined.

Once the genome had been syntenically ordered, a gene presence/absence matrix of different isolates aligned against each other made regions of dynamic gene gain and loss readily apparent (Supplementary Fig. 6). To determine whether these regions corresponded to the known mobile genetic elements present in *S. suis*, a literature search was carried out for these elements. In addition, each of the completed genome sequences was manually inspected for putative prophage and genomic islands. A prophage was defined by the presence of gene clusters related to the synthesis of phage structural proteins (that is, capsid, packaging and tail), phage replication, lytic and lysogenic life cycles. The genomic islands (GIs) were selected as regions with CDSs encoding restriction and modification enzymes and other mobility-related proteins. Each of the CDSs of these elements were BLASTed against a database consisting of a single representative of each homology group in the pan genome, and regions with significant BLAST hits are shown in Supplementary Fig. 6. To detect unknown mobile genetic elements, regions containing prophage genes and transposases (as defined by their Prokka annotations) were highlighted. These are scored as MGEs detected by annotation. To detect unknown plasmid sequences, homology groups that did not appear in the pan genome were inspected manually. Two previously unknown plasmids were identified in this way.

### Detecting population genetic structure

The population structure was estimated with BAPS (Bayesian Analysis of Population Structure) software v6.0 (refs 45, 46), in particular, its hierBAPS module^47^, which fits lineages to genome data using nested clustering. BAPS has been shown to efficiently estimate bacterial population structure from both limited core genome variation^8^,^45^,^48^ and from whole-genome sequence data^49^,^50^,^51^. Three nested levels of molecular variation were fitted to the core genome alignments of 390 and 459 isolates, respectively. The estimation used 10 independent runs of the stochastic optimization algorithm with the *a priori* upper bound of the number of clusters varying over the interval 50–200 across the runs. For the 390 isolate alignment, the estimated mode clustering had 5, 15 and 34 clusters at the levels 1–3 of the hierarchy, respectively. For the 459 isolate alignment, the estimated mode clustering had 5, 16 and 40 clusters at the levels 1–3 of the hierarchy, respectively. All clusters in both mode estimates were significantly supported when compared awith alternative partitions (posterior probability of any cluster at least 100-fold higher than for the alternative).

### Analysis of recombination

The BratNextGen method^52^ was used to detect recombination events using the core sequence alignment for the 390 isolates. The estimation of recombination was performed with the hidden Markov model hyperparameter alpha set to 1 and using 20 iterations of the estimation algorithm, which was assessed to be sufficient, as changes in the hidden Markov model parameters were already negligible over the last 40% of the iterations. Significance of a recombining region was determined as in ref. 52 using a permutation test with 100 permutations executed in parallel on a cluster computer with a threshold of 5% to conclude significance for each region. Each significant recombinant region was then masked as missing data in the alignment to obtain input data for reliable estimation of the phylogeny of the isolates. The recombination relative to point mutation (*r/m* values) was obtained using the BratNextGen output according to the same method given in ref. 49.

### Molecular dating

To date the origin of the zoonotic clade, raw reads from all isolates identified as belonging to population 1 from the BAPS analysis were mapped to the genome BM407. In addition, we included raw reads from isolate P1/7 and SC84, which were publically available. SNPs in regions associated with MGEs, which had previously been identified by comparative genomic analysis^36^, were removed. In addition, SNPs in regions that were predicted to have arisen by homologous recombination were identified using Gubbins^53^.

We produced a dated phylogeny of these isolates using the Bayesian software package BEAST v1.8.0 (ref. 54). We used an uncorrelated lognormal model of rate evolution, with rate estimated from the dated tip method and the HKY85 model of sequence evolution with a gamma distribution of rates. To estimate the relative node ages, we compared an exponential coalescent tree prior model with a constant growth tree prior model, calculating the Bayes Factor of the different models using the harmonic mean estimator of the marginal likelihood with 10,000 bootstraps. For the two models, we ran two independent MCMC until convergence was reached and the burn-in represented <10% of the chain. Convergence of all parameters was checked in the program Tracer v1.4 (ref. 55).

### Genome-wide associations

We used Discriminant Analysis of Principle Components (DAPC) 11 implemented in the R package *adegenet*^56^,^57^, to determine whether the genotypes of the isolates (both SNPs in the core genome and accessory genes) were distinct between different hosts (pig/human) and isolates of different clinical phenotypes (non-clinical/respiratory/systemic). DAPC identifies linear combinations of SNPs and presence/absence of accessory genes that best discriminate between these different groups. We retained only SNPs with a >1% frequency of the minor allele to alleviate the influence of sequencing errors (which will appear as single variants) on the analysis. We retained 80% of the total genetic variation of the principle components and kept all discriminant functions. We also repeated the analysis keeping 70 and 90% of the genetic variation to check consistency and results were qualitatively unchanged.

The frequencies of different genes in different subsets of the isolates (that is, different countries, different clinical phenotypes) were all determined from a presence/absence gene matrix defined by the orthoMCL analysis, using custom-built R scripts^58^. To perform an association analysis of human adaptation, including both core and accessory genomic elements, we used the distributed string mining method^22^. The method was used to scan *k*-mers (DNA words of length *k*) with all values of *k* between 10 and 100 from each assembled genome in BAPS cluster 1 and to store them in a sparse matrix indicating presence/absence of each *k*-mer in each isolate (191 isolates in total). The total number of distinct *k*-mers present in the genomes was 3,237,877. An association analysis was then done to detect whether any *k*-mers were significantly enriched among isolates from human hosts. To reduce the set of *k*-mers, we first filtered out all words not leading to a *P* value <5% in a standard *χ*^2^-test of association with the human host. The more detailed association analysis was then conducted with the remaining 125,593 *k*-mers using logistic regression and permutation tests for significance as in standard software such as PLINK^21^. To account for the population structure, second level BAPS clusters nested within cluster 1 were included as a covariate in the logistic regression model and permutation tests were based on 1,000,000 random permutations of the host of each isolate (human/pig). An association was determined significant if a *k*-mer had a *P*-value<0.05/125,593.

To test for evidence of human adaptation in the core genome, we also used the Cochran–Mantel–Haenszel (CMH) test in the software PLINK using a bonferroni correction for multiple tests. To control for bacterial relatedness creating spurious associations, we used the BAPs level 3 clustering in the CMH test and also mapped any significant single-nucleotide polymorphisms (SNPs) on to the recombination-stripped phylogeny and discarded any SNP that evolved only once (as inferred from parsimony).

## Additional information

**Accession codes:** The raw sequence reads generated from Illumina High-Throughput sequencing have been deposited in the Sequence Read Archive database under the accession codes ERS018463, ERS132352 to ERS132367, ERS132370 to ERS132376, ERS132380, ERS132382 to ERS132404, ERS132406, ERS132409 to ERS132454, ERS132456 to ERS132469, ERS132471 to ERS132500, ERS132502, ERS132505 to ERS132536, ERS132538 to ERS132551, ERS156197 to ERS156218, ERS156220 to ERS156290 and ERS156292 to ERS156388. Genome assemblies have been deposited in the European Nucleotide Archive under the accession codes ERS655506 to ERS655880, with study accession number PRJEB8392.

**How to cite this article:** Weinert, L. A. *et al.* Genomic signatures of human and animal disease in the zoonotic pathogen *Streptococcus suis*. *Nat. Commun.* 6:6740 doi: 10.1038/ncomms7740 (2015).

## Supplementary Material

Supplementary Information Supplementary Figures 1-10
