# Publisher Correction: Genomic signatures of human and animal disease in the zoonotic pathogen *Streptococcus suis*

**DOI:** 10.1038/s41467-019-13138-w

**Published:** 2019-11-22

**Authors:** Lucy A. Weinert, Roy R. Chaudhuri, Jinhong Wang, Sarah E. Peters, Jukka Corander, Thibaut Jombart, Abiyad Baig, Kate J. Howell, Minna Vehkala, Niko Välimäki, David Harris, Tran Thi Bich Chieu, Nguyen Van Vinh Chau, James Campbell, Constance Schultsz, Julian Parkhill, Stephen D. Bentley, Paul R. Langford, Andrew N. Rycroft, Brendan W. Wren, Jeremy Farrar, Stephen Baker, Ngo Thi Hoa, Matthew T. G. Holden, Alexander W. Tucker, Duncan J. Maskell, BRaDP1T Consortium, Janine T. Bossé, Yanwen Li, Gareth A. Maglennon, Dominic Matthews, Jon Cuccui, Vanessa Terra

**Affiliations:** 1 Department of Veterinary Medicine, University of Cambridge, Cambridge, CB3 0ES UK; 2 Department of Molecular Biology and Biotechnology, University of Sheffield, Sheffield, S10 2TN UK; 3 Department of Mathematics and Statistics, University of Helsinki, Helsinki, 00100 Finland; 4 MRC Centre for Outbreak Analysis and Modelling, Department of Infectious Disease Epidemiology, Faculty of Medicine, London, W2 1PG UK; 5 Department of Computer Science, University of Helsinki, Helsinki, 00100 Finland; 6 The Wellcome Trust Sanger Institute, Wellcome Trust Genome Campus, Hinxton, Cambridge CB10 1SA UK; 7 Oxford University Clinical Research Unit, Wellcome Trust Major Overseas Programme, Hospital for Tropical Diseases, Quan 5, Ho Chi Minh City, Vietnam; 8 Hospital for Tropical Diseases, Quan 5, Ho Chi Minh City, Vietnam; 9 Centre for Tropical Medicine, Nuffield Department of Medicine, University of Oxford, Oxford, OX3 7LJ UK; 10 Department of Global Health—Amsterdam Institute for Global Health and Development, Academic Medical Centre, University of Amsterdam, Amsterdam, 1100 DE The Netherlands; 11 Section of Paediatrics, Faculty of Medicine, Imperial College London, London, W2 1PG UK; 12 The Royal Veterinary College, Hawkshead Campus, Hertfordshire, AL9 7TA UK; 13 Faculty of Infectious & Tropical Diseases, London School of Hygiene & Tropical Medicine, London, WC1E 7HT UK; 14 Present Address: Department of Medicine, University of Cambridge, Addenbrookes Hospital, Hills Road, Cambridge, CB2 0SP UK; 15 Present Address: The Wellcome Trust, Gibbs Building, 215 Euston Road, London, NW1 2BE UK; 16 Present Address: School of Medicine, University of St. Andrews, St Andrews, KY16 9TF UK

Correction to: *Nature Communications* 10.1038/ncomms7740, published online 31 March 2015.

The Supplementary Data 1-5 files associated with this Article are corrupted. The correct versions of Supplementary Data 1-5 can be found as Supplementary Information associated with this Correction. The error has not been corrected in the Supplementary Information associated with this Article.

## Supplementary Information

Table S1

Table S2

Table S3

Table S4

Table S5

