# Supplementary material for: Genomic signatures of human and animal disease in the zoonotic pathogen Streptococcus suis
Source: Nat Commun. 2015 Mar 31;6:6740. doi: 10.1038/ncomms7740 (PMC4389249; doi:10.1038/ncomms7740)
Supplement: Supplementary Information — Supplementary Figures 1-10 [file ncomms7740-s1.pdf]

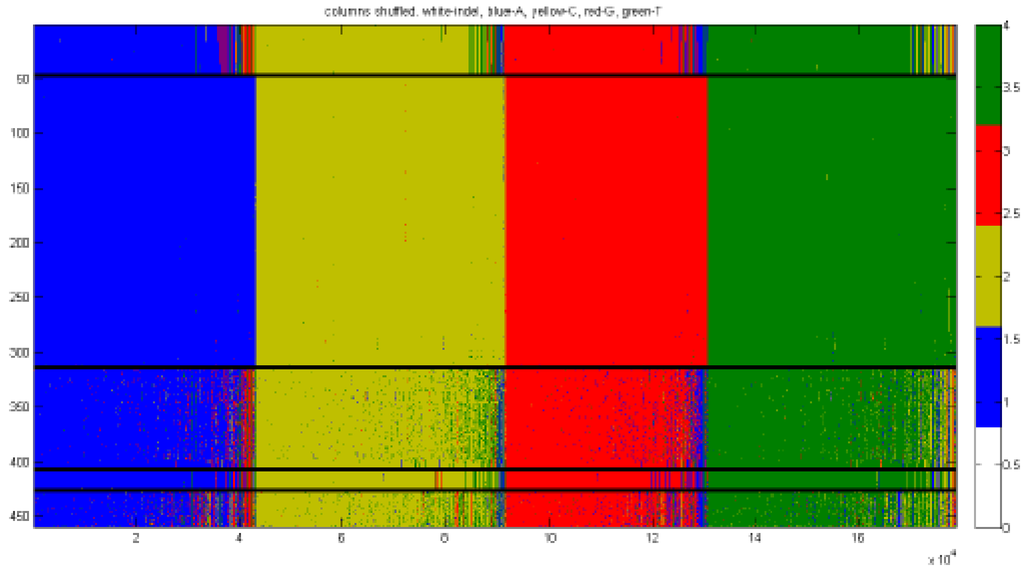


**Supplementary Figure 1. Bayesian Analysis of Population Structure of 459 global isolates of *S. suis***

Each row in the figure is a strain and each column is a SNP (ordered to maximize visual separation of the five distinct clusters) and horizontal black lines are drawn to separate the five clusters. The colour pattern of these SNPs within the black lines is consistent with them being assigned to the same BAPs group.

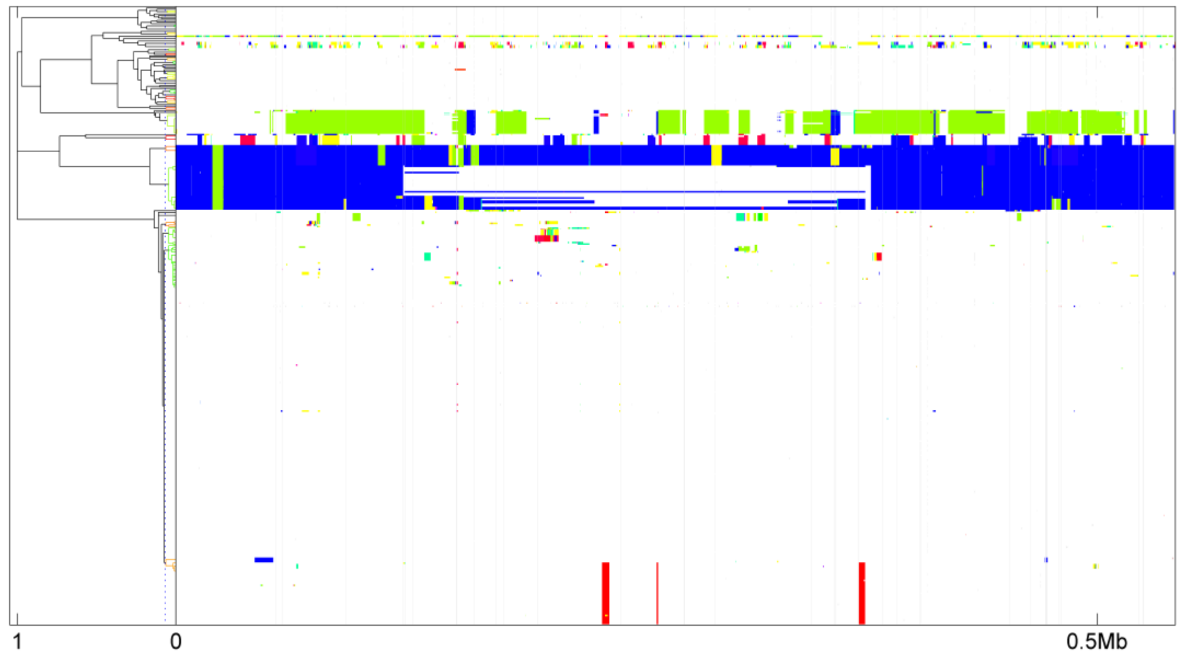


**Supplementary Figure 2. Bayesian Analysis of Recombination of 390 isolates from the UK, Vietnam and published whole genomes.**

Figure showing the estimated recombination events from the BRAT analysis. Each strain is a row in the figure and the colours refer to putative recombination blocks with the shade of colour indicating which of the donor clusters is most likely to have been the origin of the sequence. Some isolates have experience recombination throughout most of their genome (large blue blocks) whereas some isolates (no colour) look more clonal.

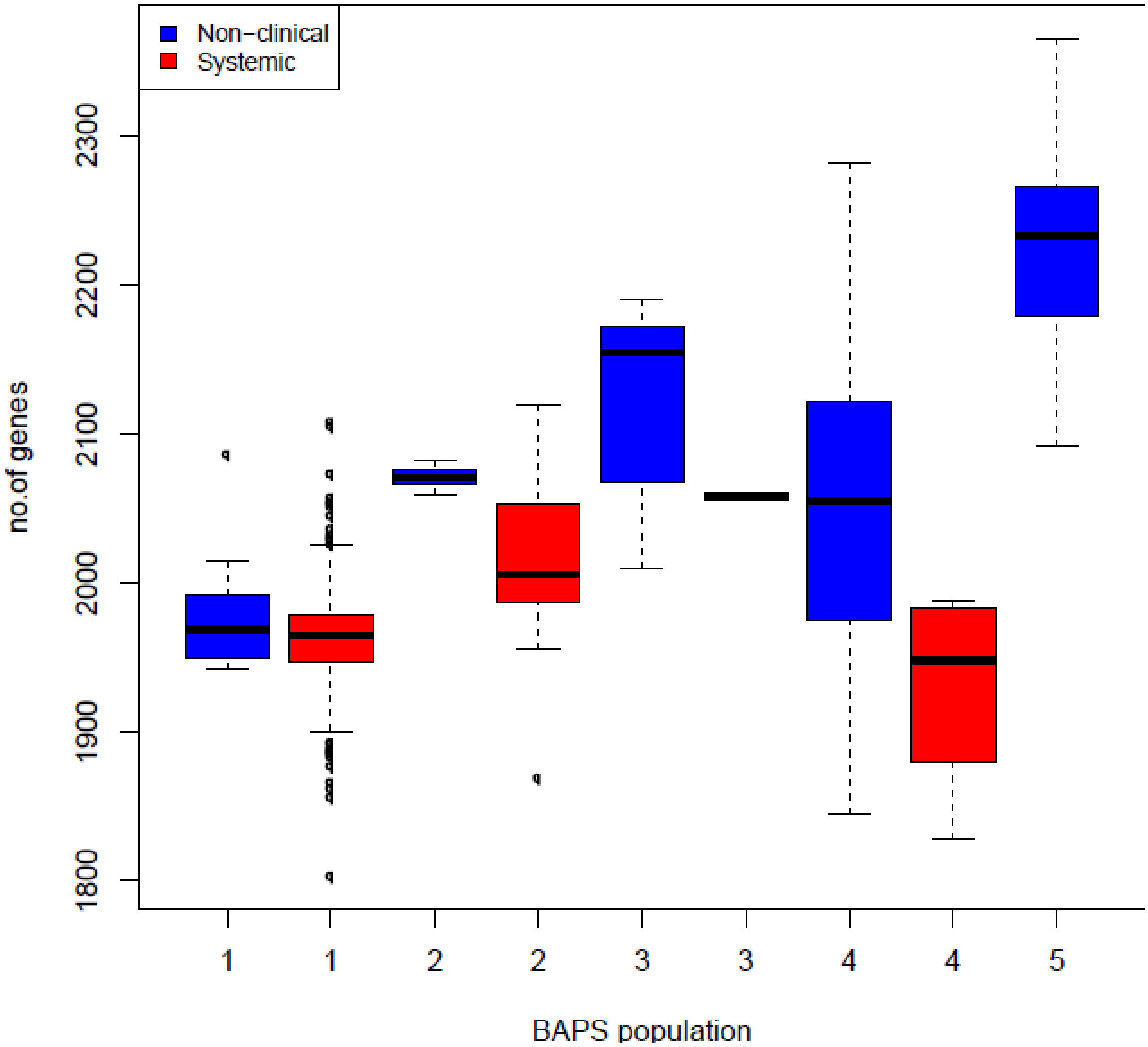


**Supplementary Figure 3. Genome size difference between systemic and non-clinical isolates in the different BAPS populations.**

Populations 2-4 show a consistent decline in gene number between systemic and non-clinical isolates. Population 1 (which is made up of mostly clinical isolates) has around 200 genes less than Population 5 (which is made up of mostly non-clinical isolates). Boxes show the medians and upper and lower quartiles; whiskers show the most extreme values within 1.5 times the interquartile range. Sample sizes within Population 1 non-clinical = 34 systemic = 208, Population 2 non-clinical = 11 systemic = 9, Population 3 non-clinical = 8 systemic = 1, Population 4 non-clinical = 31 systemic = 6, Population 5 non-clinical = 10.

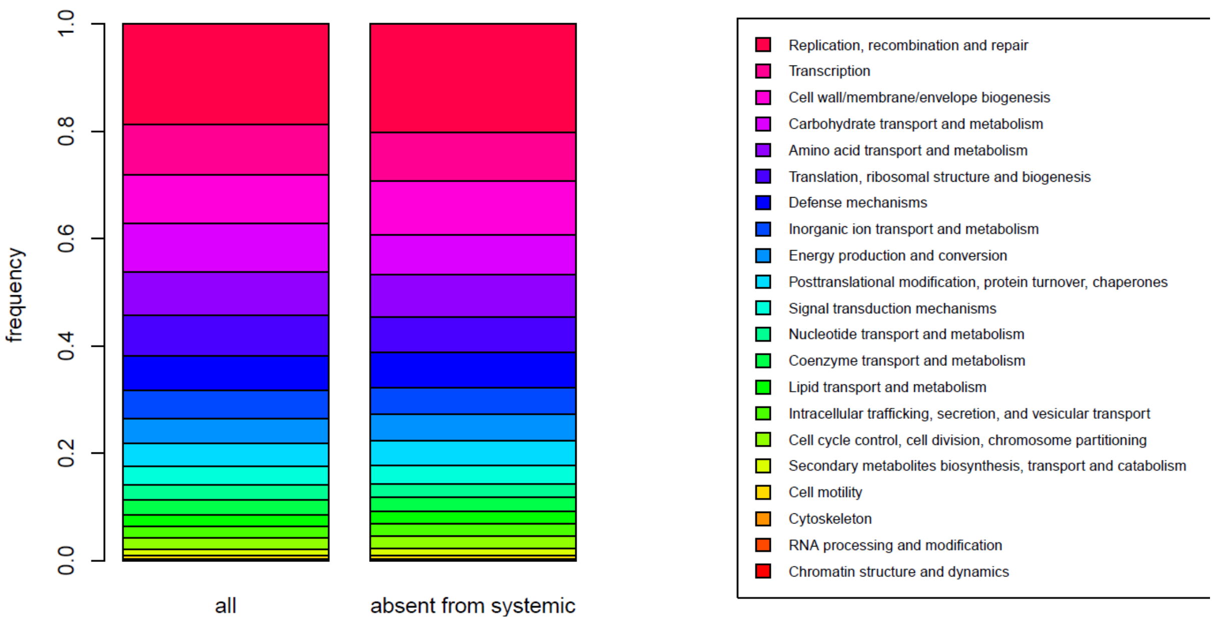


**Supplementary Figure 4. COG functional breakdown of genes present vs. genes completely absent in systemic isolates.**

There is no difference in the distribution of functional classes between genes that are completely absent in systemic isolates, and those that were present.

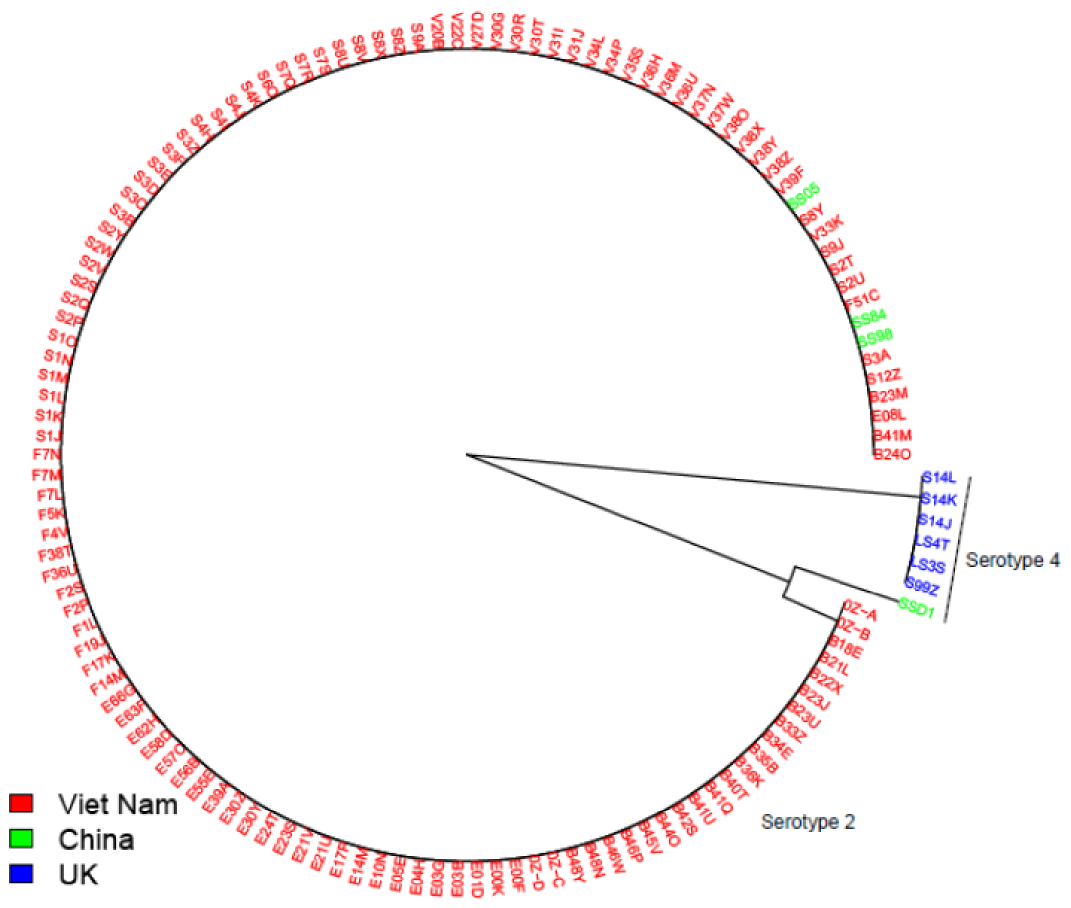


**Supplementary Figure 5. Neighbor-joining tree of *salK* from all of the isolates from the 390 dataset.**

The phylogeny shows that the *salK* gene in serotype 2 isolates from Vietnam and China is divergent from the *salK* gene in serotype 4 isolates from China and the UK.

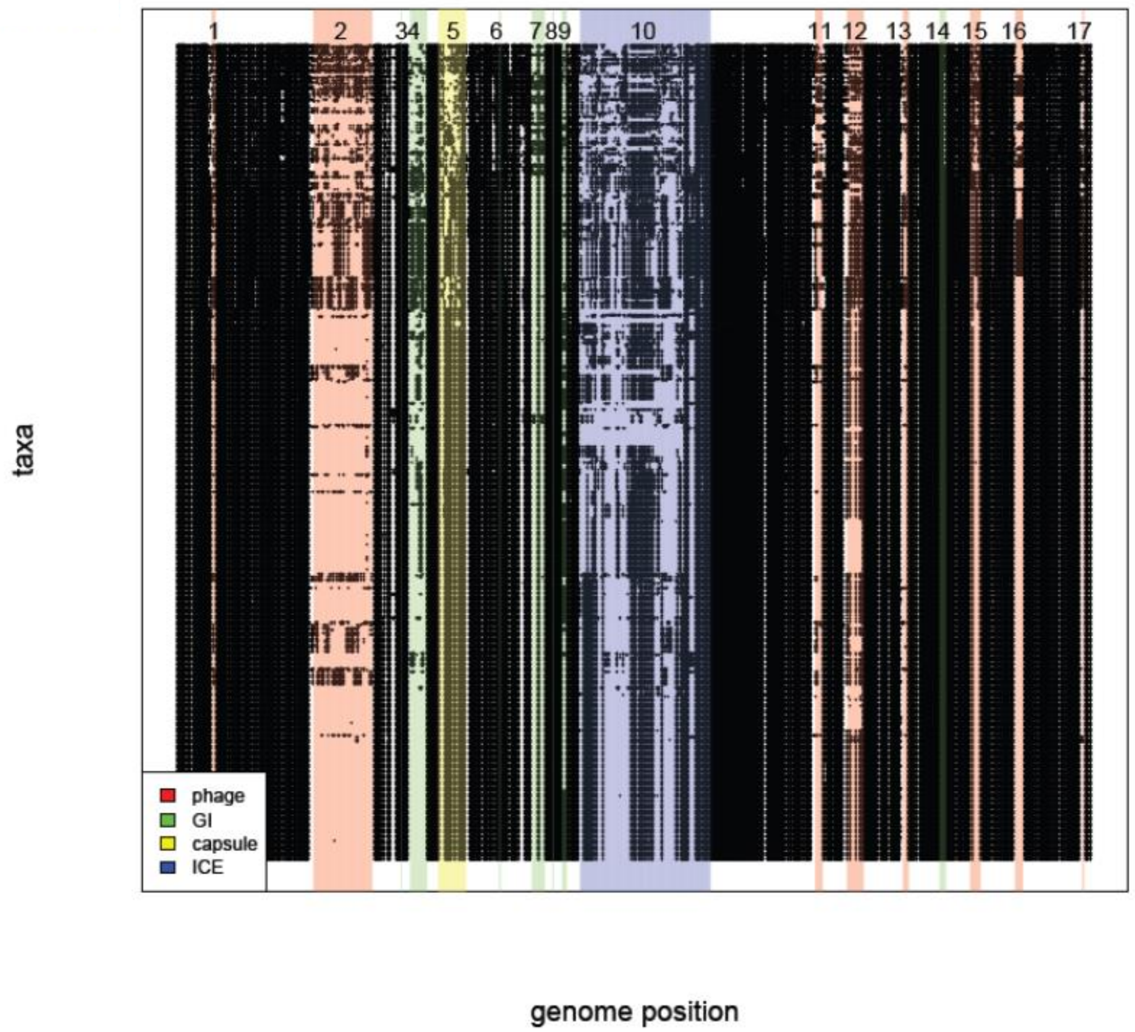


**Supplementary Figure 6. Pan genome of *S. suis* showing presence/absence of genes in different isolates and locations of 17 MGEs.**

Each row in the Figure is a strain and each column a gene. Presence of gene is indicated in black, absence in white and this shows regions of the pan genome undergoing dynamic gene and gain loss corresponding to MGEs. The annotation of MGEs was defined in two ways, by BLASTing known MGEs to the pan genome and identifying the linked regions, or by their Prokka annotations (i.e. phage proteins, transposases). In this way 17 MGEs were identified. The numbers at the top of the figure correspond to 1) Phage 1 identified by annotations, 2) Phage 2 identified by BLAST (the only known active lytic phage), 3) genomic island (GI) 1 identified by BLAST, 4) GI2 identified by annotations, 5), GI3 identified by BLAST 6), GI4 identified by annotations 7), GI5 identified by BLAST 8), GI6 identified by BLAST 9), GI7 identified by BLAST, 10) Integrative Conjugative Element (ICE) 11), Phage 3 identified by annotations, 12), Phage 4 identified by BLAST, 13), Phage 5 identified by BLAST 14), GI8 identified by BLAST, 15), Phage 6 identified by annotations 16), Phage 7 identified by annotations 17) Phage 8 identified by annotations

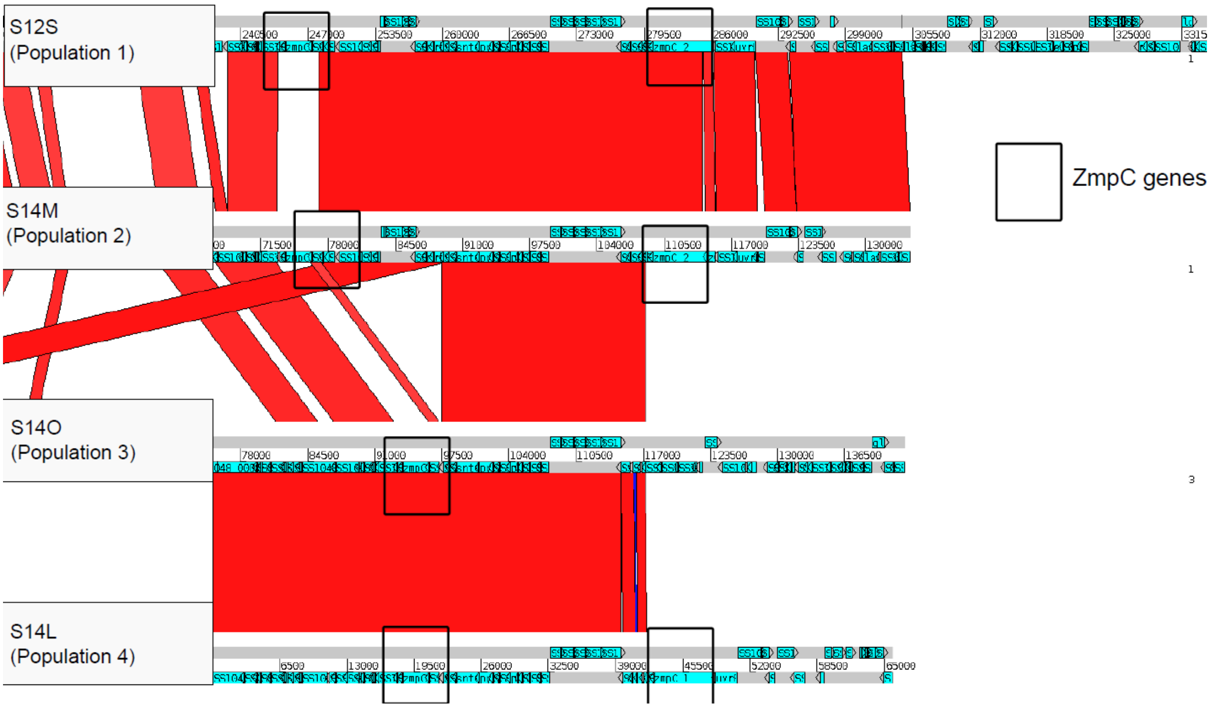


**Supplementary Figure 7. Comparison of the region of the ICE with** an abundance of IgA-specific zinc metalloproteinases **from populations 1-4.**

This region is consistently over-represented in respiratory isolates and contains a type IV secretion system as well as an abundance of IgA-specific zinc metalloproteinases.

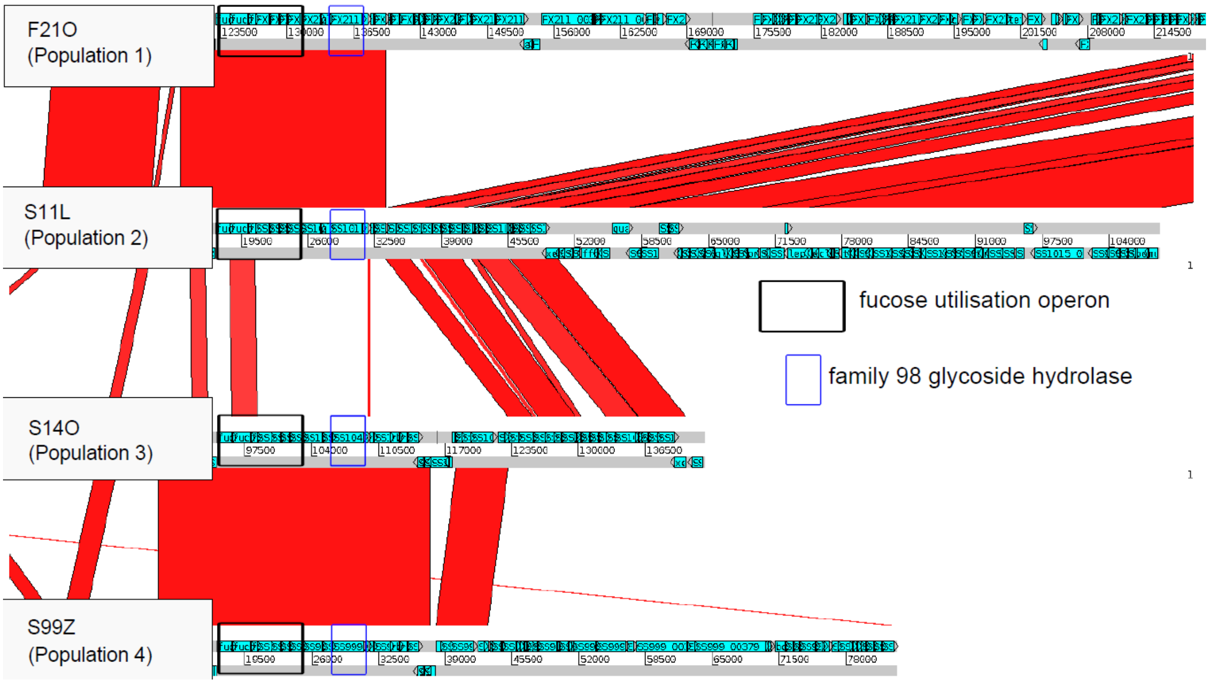


**Supplementary Figure 8. Comparison of the** fucose utilisation operon **region of the ICE from populations 1-4.**

This region is consistently over-represented in respiratory isolates and contains a fucose utilisation operon with a virulence factor belonging to a family 98 glycoside hydrolase.

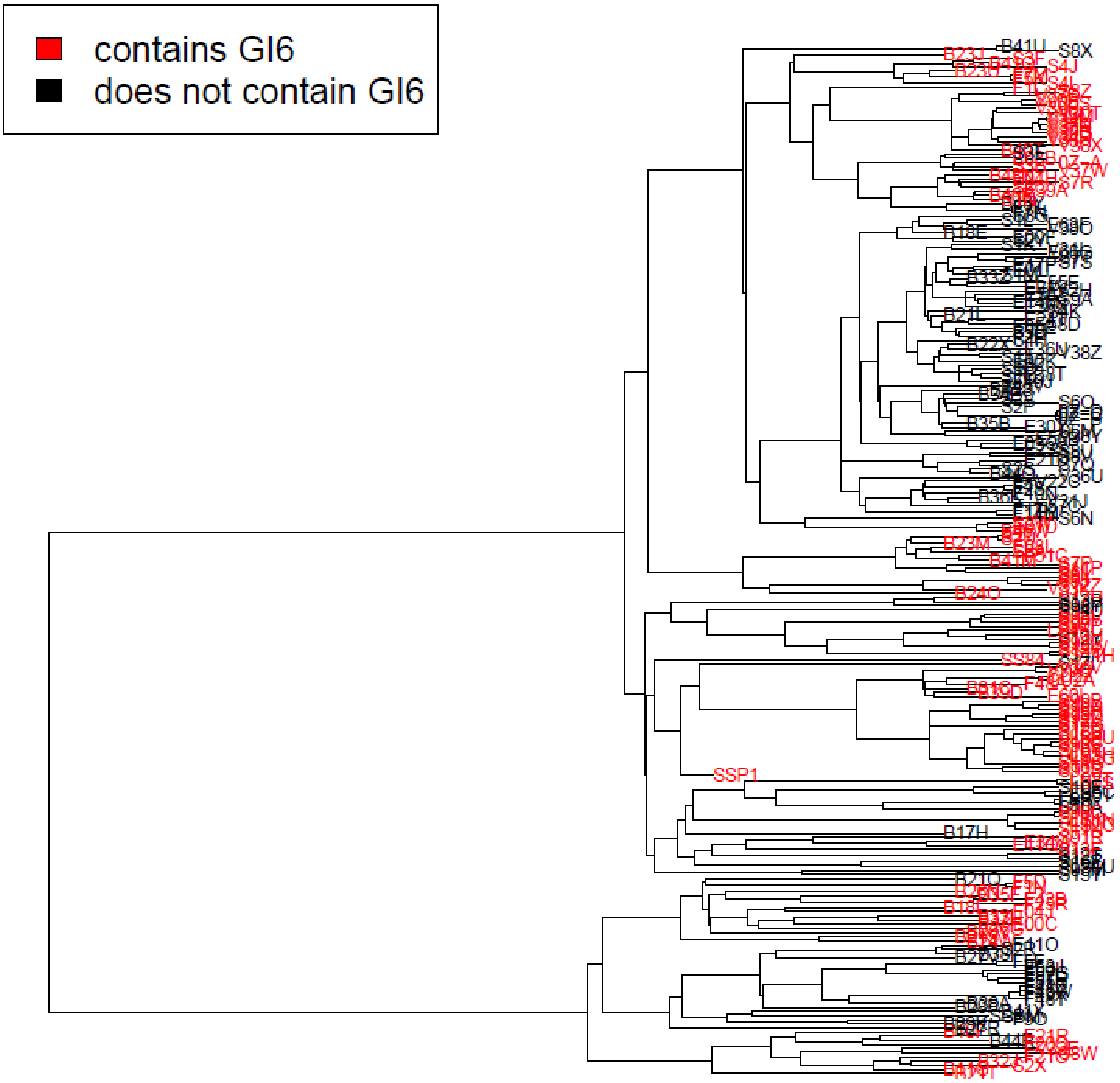


**Supplementary Figure 9. Dated phylogeny of 256 isolates of *S. suis* from the virulent population one showing presence of absence of genomic island 6.**

The lack of this genomic island explains the discrimination seen between human and pig populations in Vietnam (Fig. 6), but many human isolates found also lack this genomic island.

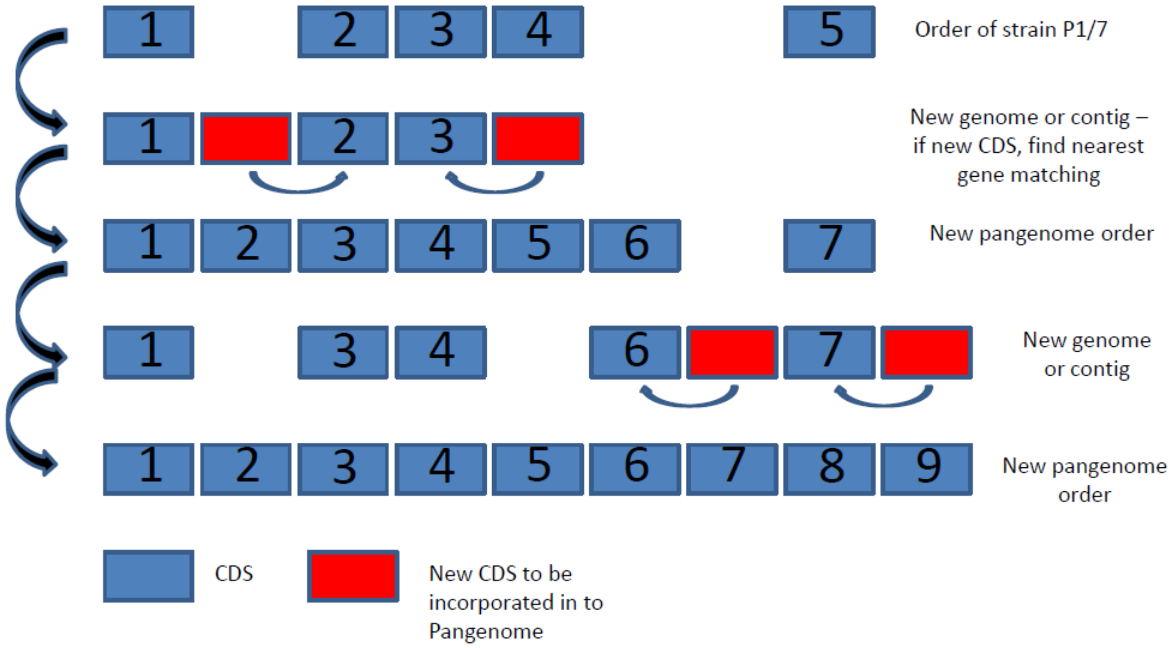


**Supplementary Figure 10. Algorithm used to create the syntenic pan genome.**
